# Checkpoint inhibitors in hematological malignancies

**DOI:** 10.1186/s13045-017-0474-3

**Published:** 2017-05-08

**Authors:** Chi Young Ok, Ken H. Young

**Affiliations:** Department of Hematopathology, The University of Texas MD Anderson Cancer Center, 1515 Holcombe Boulevard, Houston, TX 77030-4009 USA

**Keywords:** PD-1, PD-L1, PD-L2, CTLA-4, Immune checkpoint, Hematologic malignancies

## Abstract

Inhibitory molecules such as PD-1, CTLA-4, LAG-3, or TIM-3 play a role to keep a balance in immune function. However, many cancers exploit such molecules to escape immune surveillance. Accumulating data support that their functions are dysregulated in lymphoid neoplasms, including plasma cell myeloma, myelodysplastic syndrome, and acute myeloid leukemia. In lymphoid neoplasms, aberrations in 9p24.1 (*PD-L1*, *PD-L2*, and *JAK2 locus*), latent Epstein-Barr virus infection, *PD-L1* 3′-untranslated region disruption, and constitutive JAK-STAT pathway are known mechanisms to induce PD-L1 expression in lymphoma cells. Clinical trials demonstrated that PD-1 blockade is an attractive way to restore host’s immune function in hematological malignancies, particularly classical Hodgkin lymphoma. Numerous clinical trials exploring PD-1 blockade as a single therapy or in combination with other immune checkpoint inhibitors in patients with hematologic cancers are under way. Although impressive clinical response is observed with immune checkpoint inhibitors in patients with certain cancers, not all patients respond to immune checkpoint inhibitors. Therefore, to identify best candidates who would have excellent response to checkpoint inhibitors is of utmost importance. Several possible biomarkers are available, but consensus has not been made and pursuit to discover the best biomarker is ongoing.

## Background

The hallmarks of cancer are constant proliferative signaling, evasion of growth suppressors, resistance to cell death, replicative immortality, induction of angiogenesis and activating invasion, and metastasis [1]. The dysregulated cellular processes in cancer cells are in tandem with accumulation of variable genetic alterations and consequent expression of tumor neoantigens which are not present in normal cells [2]. In ideal state, immune cells recognize these new antigens and kill the cancer cells. The whole process is elegantly explained by Chen and Mellman with the concept of the cancer-immunity cycle, which consists of several steps [3]. Firstly, dissemination of cancer neoantigens to tumor microenvironment (TME) occurs following cancer cell death (step 1). Afterwards, the released cancer neoantigens are captured and processed by antigen presenting cells, i.e., dendritic cells, where the processed neoantigens are presented as a complex with major histocompatibility complex (MHC) I or II molecules (step 2). The following step is priming and activation of effector T cell against the cancer neoantigens (step 3). Owing to higher density of antigen presenting cells in lymphoid organs, the second and third steps mostly occur in peripheral lymphoid organs. Following the priming and activation, the activated effector T cells then migrate to the tumor site via blood vessels (step 4). When the activated effector T cells arrive in the vicinity of the tumor site, they pass through endothelial cells and infiltrate the tumor microenvironment (step 5). Once successfully infiltrated, the activated effector T cells bind cancer cells recognizing cancer neoantigens presented on MHC I molecule (step 6). Finally, the activated effector T cells induce apoptosis of the cancer cells by releasing cytotoxic molecules including granzyme or perforin via Fas-Fas ligand interaction (step 7). Oftentimes, cancer cells or immunosuppressive cells in the TME provide immune inhibitory signals lest effector T cells function properly.

In the context of the cancer-immunity cycle, checkpoint inhibitors aim to reset or reinstate dysfunctional effector T cells. Clinical studies using checkpoint inhibitors have shown significant responses in various cancers [4–7]. In this review, we discuss biology of immune inhibitory molecules, their roles in hematological cancers, different types of checkpoint inhibitors, clinical trials on patients with hematologic cancers, and search for biomarkers in checkpoint inhibitor therapy.

## Maint text

### Biology of immune inhibitory molecules

**Fig. 1:**
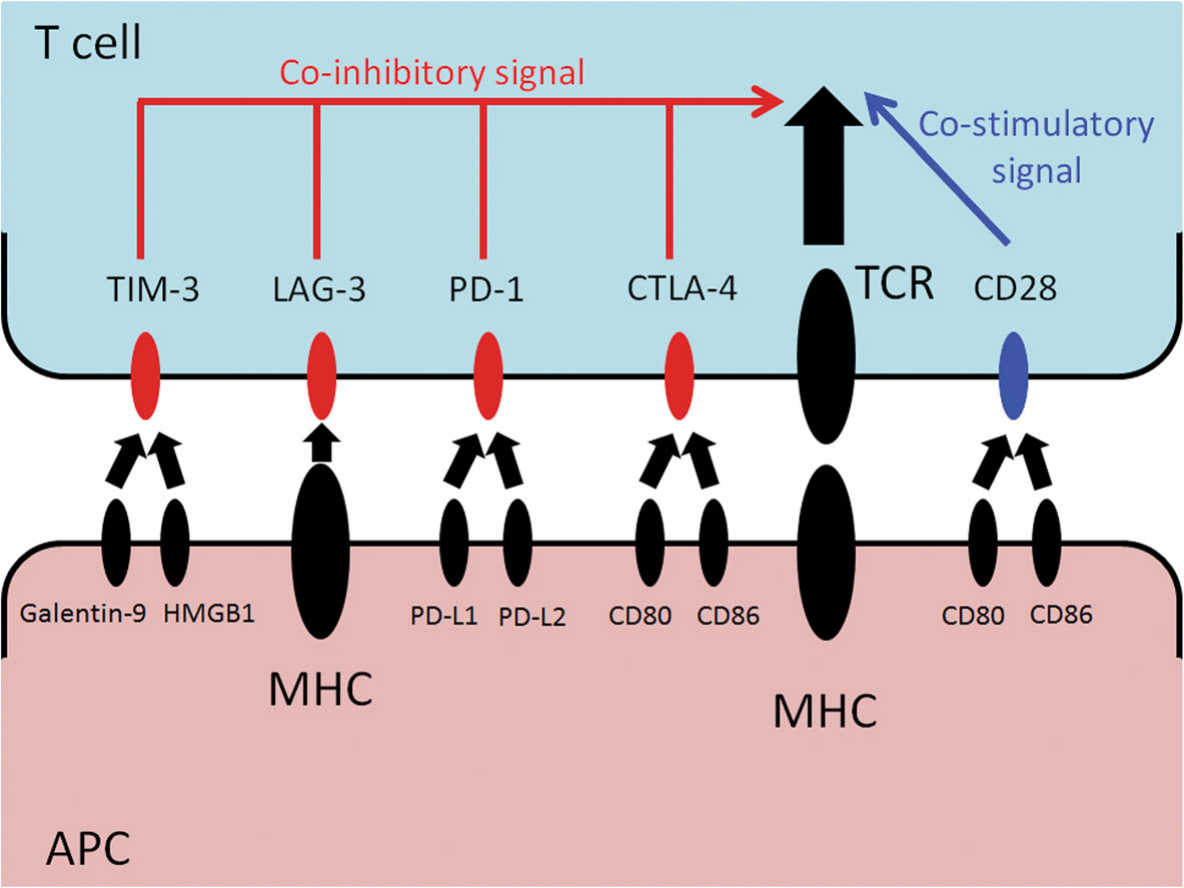
T cell activation signals. The main signal is mediated by T cell receptor. Co-stimulatory signal is provided by CD28. Co-inhibitory signals are mediated by CTLA-4, PD-1, LAG-3, or TIM-3. *TIM-3* T cell immunoglobulin and mucin domain-containing protein-3. *LAG-3* lymphocyte activation gene-3, *PD-1* programmed death-1, *CTLA-4* cytotoxic T-lymphocyte antigen-4, *TCR* T cell receptor, *HMGB1* high mobility group protein B1, *MHC* major histocompatibility complex, *PD-L1* programmed death-ligand 1, *PD-L2* programmed death-ligand 2

For proper T cell activation, two separate signals are required (Fig. 1) [8]. The first signal is mediated by antigen-dependent T cell receptor (TCR) binding to the major histocompatibility complex (MHC) molecule of an antigen-presenting cell (APC). The second signal is antigen-independent, co-stimulatory, or co-inhibitory signal delivered by the APCs. The second signal modulates TCR signaling and determines the T cell’s fate. Several co-stimulatory or co-inhibitory molecules on T cells with their respective ligands are collectively known as B7-CD28 family. The prototypical co-stimulatory molecule is CD28 on resting naïve T cells, which induces cell-cycle progression, interleukin-2 (IL-2) production, and clonal expansion is constitutively expressed in resting naïve T cells [9]. Without co-stimulatory second signals, T cells fall into anergy. On the other hand, cytotoxic T-lymphocyte antigen-4 (CTLA-4) is a co-inhibitory receptor on T cells that induces T cell tolerance [10]. Additional second signal molecules include programmed death-1 (PD-1), lymphocyte activation gene-3 (LAG-3, CD223), T cell immunoglobulin and mucin domain-containing protein-3 (TIM-3), T cell immunoreceptor with immunoglobulin and ITIM domains (TIGIT), or B- and T-lymphocyte attenuator (BTLA). In this review, we discuss biology of CTLA-4, PD-1, LAG-3, and TIM-3, dysregulation of these molecules in hematologic malignancies, clinical trials, and biomarkers.

### Cytotoxic T-lymphocyte antigen-4 (CTLA-4)

**Fig. 2:**
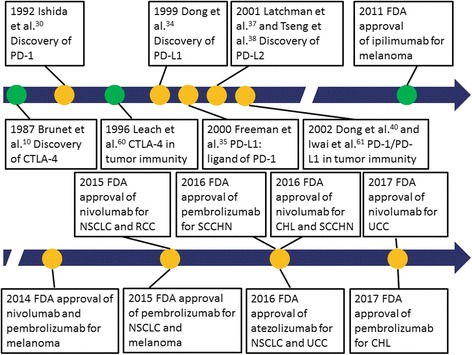
From discovery for immunocheckpoints to FDA approval of immunocheckpoint inhibitors. *CHL* classical Hodgkin lymphoma, *NSCLC* non-small cell lung cancer, *RCC* renal cell carcinoma, *SCCHN* squamous cell carcinoma of the head and neck, *UCC* urothelial carcinoma

CTLA-4 (also known as CD152) was first discovered by Brunet et al. (Fig. 2) [10]. It is a protein encoded by the 4-exon *CTLA4* gene on chromosome 2q33.2. It belongs to the immunoglobulin superfamily, with a single immunoglobulin V-like domain containing ligand binding sites [10, 11]. It consists of 223 amino acids, and with a calculated molecular weight of 24.6 kDa. CTLA-4 mainly resides in the cytoplasm in naïve resting T cells, but its expression on the surface of T cells can be detected within 1 or 2 days after activation [12]. On the other hand, rapid induction of CTLA-4 expression is seen in memory T cells upon activation, and its expression lasts longer compared with naïve resting T cells [13]. In regulatory T cells, CTLA-4 is constitutively expressed [14].

Although their functions are opposite, CLTA-4 and CD28 share the same ligand, B7-1 and B7-2. They share the MYPPPY motif for ligand binding [15]. Of note, CTLA-4 expression is 30- to 50-fold less than that of CD28 even in its maximum state upon activation. However, the affinity and avidity for CTLA-4 and its ligands are much greater than CD28 because the former homodimerizes and can bind to B7 molecules bivalently [16]. Upon activation by ligand binding, CTLA-4 molecules migrate from the cytoplasm to the cell surface, and this migration is dependent on the strength of T cell receptor signaling and phosphorylation of the Y^165^VKM motif in the cytoplasmic domain of CTLA-4 [17–20]. Furthermore, redistribution of CTLA-4 to the immunological synapse was shown to be highly dependent on B7-1, but only slightly dependent on B7-2 [21].

T cell inactivation by CTLA-4 can be explained by two mechanisms. Once redistribution of CTLA-4 to the proximity of immunological synapse occurs, it can sequester B7-1/B7-2 owing to its higher avidity and affinity so that the CD28-mediated co-stimulatory signal would be reduced (competitive antagonism) [22]. The second mechanism is for CTLA-4 to deliver an inhibitory signal via the cytoplasmic tail. Although the precise mechanism is not unequivocally determined, CTLA-4 signal inhibits nuclear accumulation of activator protein 1 (AP-1), NF-κB, and nuclear factor of activated T cells (NFAT) in activated T cells [23, 24]. Furthermore, CTLA-4 halts cell cycle progression by direct inhibition of cyclin-dependent kinase 4 (CDK4), CDK6, and cyclin D3 [25]. CTLA-4 also selectively inactivates microtubule-associated protein kinase (MAPK), extracellular signal-regulated kinase-1 (ERK), and c-Jun NH2-terminal kinase (JNK), which are required for stimulation of IL-2 production [26].

The cytoplasmic tail of CTLA-4 does not contain an immune receptor tyrosine-based inhibitory motif (ITIM) and does not have intrinsic enzymatic activity. Instead, CTLA-4 inhibitory effects (phosphatase activity) are thought to be mediated with other molecules including serine/threonine phosphatase PP2A and/or Src homology 2 domain-containing phosphatases (SHPs). PP2A is bound to newly synthesized CTLA-4 molecules and makes CTLA-4 inactive [27]. Upon ligand binding in the vicinity of TCR, the scaffolding subunit of PP2A is phosphorylated and PP2A is dissociated from CTLA-4. The dissociated PP2A inhibits the phosphatidylinositol 3-kinase (PI3K)/Akt pathway via directly inactivating protein kinase B/Akt [28]. In addition, Guntermann and Alexander demonstrated that the majority of phosphatase activity of CTLA-4 was attributed to SHP-1 [29]. Because CTLA-4 lacks ITIM, which is a direct binding site of SHP-1, it is thought that adapter proteins might be needed for interaction between CTLA-4 cytoplasmic domains and SHP-1.

### Programmed death-1 (PD-1)

PD-1 (also known as CD279) was first discovered by Ishida et al. from Tasuku Honjo’s group in 1992 in search of a gene inducing apoptosis [30]. PD-1 is a transmembrane protein with 288 amino acids and is encoded by *PDCD1* gene on chromosome 2q37.3. PD-1 contains a single immunoglobulin V-like domain, a transmembrane domain, and an intracellular domain. The intracellular domain has an ITIM (S/I/V/LxYxxI/V/L) and an immunoreceptor tyrosine-based switch motif (ITSM; TxYxxV/I) [31, 32]. Expression of PD-1 is present in effector T cells, regulatory T cells (Treg), naïve and activated B cells, natural killer cells, myeloid dendritic cells, and monocytes with low intensity. In resting T cells, PD-1 expression is not present, but it can be induced within 24 h of T cell activation [33].

Programmed death-ligand 1(PD-L1) and programmed death-ligand 2 (PD-L2) are ligands for PD-1. Search of ligand for PD-1 was actively sought by Dr. Honjo’s group, but PD-L1 (also known as CD274 or B7-H1) was independently discovered by Dong et al. in Lieping Chen’s group in 1999 [34]. Dr. Chen’s group identified a molecule named B7-H1, but was not aware it was actually a ligand to PD-1. 1 year later, Gordon Freeman’s group, in collaboration with Honjo’s group, discovered a ligand for PD-1 (PD-L1) and demonstrated that PD-L1 is identical to B7-H1 [35]. PD-L1 is encoded by *CD274* gene on chromosome 9p24.1. In non-pathologic lymphoid tissue, PD-L1 expression is observed in follicular T cells, macrophages, and a subset of dendritic cells. PD-L1 is also seen in placental syncytiotrophoblasts and dendritic cells/monocytes in the lung and liver [33, 34, 36]. By collaborative study in Honjo, Freeman, and Arlene Sharpe’s group, PD-L2 (also known as CD273 or B7-DC) was identified in 2001 [37]. In the same year, Tseng et al. in Drew Pardoll’s group independently discovered PD-L2 [38]. PD-L2 is encoded by *PDCD1LG2* gene on chromosome 9p24.1. Of note, *CD274* gene and *PDCD1LG2* gene are 42 kB apart from each other. Compared with PD-L1, PD-L2 expression is more restricted. It is only seen in activated CD4^+^ or CD8^+^ T cell subsets, myeloid dendritic cells, monocytes, endothelial cells, and placental syncytiotrophoblasts [39]. Expression of PD-L1 and PD-L2 can be induced by interferon gamma (IFN-γ), granulocyte macrophage colony-stimulating factor (GM-CSF), and IL-4 [37, 40–42].

PD-1 negatively regulates IL-2 production and T cell proliferation [43, 44]. Upon ligand binding, ITIM and ITSM in the cytoplasmic domain of PD-1 are phosphorylated by the Src-family tyrosine kinases and SHPs are further recruited to the phosphorylated tyrosine residue. SHPs dephosphorylate downstream signal pathways including PI3K/Akt or RAS/MEK/ERK pathway, blocking cell cycle progression [28, 45, 46]. SHPs also inactivate zeta-chain-associated protein kinase 70 (ZAP70) and protein kinase C-θ (PKC-θ), essential for T cell activation and IL-2 production, respectively [47, 48]. However, PD-1-mediated inhibitory signals can be overcome by strong T cell stimulation with CD28 or exogenous IL-2 [49].

Inhibitory function is not the only role of PD-1 pathway. Francisco and colleagues demonstrated that PD-L1 converts naïve CD4^+^/forkhead box P3 (FOXP3)- T cells to CD4^+^/FOXP3^+^ regulatory T cells (Tregs) in vitro, even without transforming growth factor beta (TGF-β) which is a well-established stimulator of Treg induction [50]. Induction and maintenance of Tregs by PD-L1 was also shown by the same group in vivo. Although this is not an inhibitory function in cellular level, PD-1 pathway enhances immune suppression by inducing immunosuppressive Tregs in the level of organism.

While CTLA-4 and PD-1 both deliver the co-inhibitory second signal, they execute their roles at different time points in the life cycle of immune response [51, 52]. CTLA-4 functions early in the life cycle of immune response during T cell priming in lymphoid organs (central checkpoint) and affects the global impact on the immune system. CTLA-4:B7-1/B7-2 interaction diminishes CD4^+^ T effector cells, increases CD4^+^ T-helper cells and enhances immunosuppressive activity of regulatory T cells, resulting in peripheral T-cell tolerance or anergy [53]. CTLA-4-deficient mice developed fatal lymphoproliferation and multiorgan autoimmunity [54, 55]. On the other hand, PD-1 plays a role in T-cell activation in peripheral tissue containing target cells (peripheral checkpoint). PD-1:PD-L1/PD-L2 interaction attenuates TCR signaling in T cells, inducing T cell exhaustion. PD-1-deficient mice developed lupus-like autoimmune disease inflammation [56, 57].

These checkpoints in immune response are often exploited in many cancers including hematologic malignancies [58, 59]. The concept that CTLA-4 blockade can be used to enhance anti-tumor activity was first shown by Leach and colleagues [60]. The role of PD-1 pathway in tumor immunity was independently shown by Dong and Iwai, promoting PD-1 blockade in cancer therapy [40, 61].

### Lymphocyte activation gene-3 (LAG-3)

Following clinical success of targeting CTLA-4 and PD-1, other co-inhibitory molecules receive more attention; LAG-3, and TIM-3. The lymphocyte activation gene-3 (LAG-3, CD223) was discovered by Triebel and colleagues in 1990 [62]. It is encoded by 8-exon *LAG3* gene, located at 12p13.31. LAG-3 has 498 amino acids and has structural similarity to CD4, containing one immunoglobulin-like V-type domain and three immunoglobulin-like C2-type domains. The intracellular domain of LAG-3 contains a unique KIEELE motif, which is essential for T cell modulation by LAG-3 [63]. Expression of LAG-3 is present in activated T cells, NK cells, activated B cells, and plasmacytoid dendritic cells [62, 64, 65]. The major ligands of LAG-3 are class II MHC molecule on APCs and liver and lymph node sinusoidal endothelial cell C-type lectin (LSECtin) on tumor cells or hepatocytes [66]. LAG-3 is a negative regulator in CD4 and CD8 T cell expansion in vitro as well as in vivo [67]. However, precise mechanisms remain to be elucidated. Co-expression of LAG-3 and PD-1 has been seen in tumor infiltrating lymphocytes (TILs) in tumor mouse models as well as human tissue, suggesting its role similar to PD-1 [68–70]. Inhibition of both PD-1 and LAG-3 showed augmented anti-tumor activity of CD8^+^ T cells compared to targeting either of them [68, 70].

### T cell immunoglobulin and mucin domain-containing protein-3 (TIM-3)

T cell immunoglobulin and mucin domain-containing protein-3 (TIM-3) was discovered by Monney and colleagues in 2002 [71]. TIM-3 is also called hepatitis A virus cellular receptor 2 (HAVCR2) and is encoded by *HAVCR2* gene. *HAVCR2* is located at 5q33.3 and consists of seven exons. TIM-3 is a transmembrane protein, containing signal peptide sequence, immunoglobulin-like V-type domain, mucin domain, and cytoplasmic tail [71]. TIM-3 expression is present in cytotoxic T cells, T helper 1 cells, regulatory T cells, NK cells, monocytes, and dendritic cells. Ligands of TIM-3 are many, including galectin-9, high mobility group protein B1 (HMGB1), and phosphatidyl serine [72, 73]. In the absence of ligands, BAT3 (HLA-B associated transcript 3) is bound to tyrosine residues in the cytoplasmic domain, forming a complex with TIM-3. Upon bindings to ligands, BAT3 is dissociated from the tyrosine residues and FYN, which can induce T cell anergy, could replace them [74, 75]. Similar to LAG-3, co-expression of TIM-3 and PD-1 was observed in CD8^+^ TILs [76, 77]. Interestingly, TILs with PD-1-/TIM-3- and showed the most severe dysfunction, compared to TILs with PD-1^+^/TIM-3- (weak dysfunction) or TILs with PD-1^+^/TIM-3^+^ (good function) [76, 78]. Although tyrosine residues in the cytoplasmic domain of TIM-3 are thought to cooperate with downstream signaling pathways, precise mechanisms are yet to be determined. Targeting TIM-3 showed significant anti-tumor activity in tumor mouse models [79]. Inhibition of both PD-1 and TIM-3 also demonstrated enhanced anti-tumor activity of CD8^+^ TILs [76].

### Aberrancies in immune checkpoint molecules in hematological malignancies

#### Lymphomas

CTLA-4 expression is upregulated in patients with peripheral T-cell lymphoma, mycosis fungoides, and Sézary syndrome, but not seen in B-cell lymphoma [80–82]. *CTLA4-CD28* rearrangement is present in a subset of patients with angioimmunoblastic T-cell lymphoma, extranodal NK/T-cell lymphoma, peripheral T-cell lymphoma, not otherwise specified, Sézary syndrome, and adult T-cell leukemia/lymphoma [83–86]. The rearrangement generates a fusion protein including the extracellular and transmembrane domains of CTLA4 and the cytoplasmic domain of CD28, which mediates activating T cell signals via AKT and MAPK pathways [84].

PD-L1 or PD-L2 expression in tumor cells would provide immune escape signals. PD-L1 expression can be induced by extrinsic signals (e.g., IFN-γ) secreted from tumor-infiltrating lymphocytes (TILs) or by intrinsic signals [4, 87]. The former can be represented by T cell-rich, histiocyte-rich large B cell lymphomas (TCHRBCLs), which is characterized by few malignant B cells in the background of dense population of CD8^+^ T cell and histiocytes [88]. Heterogeneous PD-L1 expression is usually seen in the interface between malignant B cells and inflammatory background. Of note, histiocytes adjacent to lymphoma cells also show strong PD-L1 expression in TCHRBCL, suggesting that both tumor cells and background inflammatory cells provide immune escape signals [89].

On the other hand, relatively homogenous expression of PD-L1 is present by intrinsic signals. So far, four mechanisms in intrinsic signals have been reported in lymphoid neoplasms. Firstly, copy number alterations (amplifications or gains) and/or translocations involving 9p24.1/*PD-L1*/*PD-L2* are associated with PD-L1 overexpression in tumor cells of classical Hodgkin lymphoma (CHL), primary mediastinal large B cell lymphoma (PMBL), Epstein-Barr virus (EBV)-negative primary central nervous system lymphoma (PCNSL), primary testicular lymphoma (PTL), and in a subset of diffuse large B cell lymphoma (DLBCL) [90–94]. Of note, amplification of 9p24.1 not only increases the genetic dosage of *PD-L1*/*PD-L2* but also induces *JAK2* amplification and, consequently, enhancement of Janus kinase/signal transducer and activator of transcription (JAK/STAT) signaling [90]. Because *PD-L1* has a promoter that is responsive to the JAK/STAT signaling pathway, extra signaling for PD-L1 expression is present.

Secondly, PD-L1 expression can be induced by EBV infection. EBV latent membrane protein 1 (LMP1) activates the JAK/STAT pathway and the transcription factor AP-1 [95]. The relationship between JAK/STAT pathway and PD-L1 promoter was already discussed. PD-L1 enhancer can be stimulated by AP-1 [96, 97]. In one study, PD-L1 expression is seen in all cases of EBV-positive DLBCL (EBV^+^ DLBCL) and EBV-positive immunodeficiency-related DLBCL [89]. Other EBV-associated lymphoproliferative disorders including EBV^+^ post-transplant lymphoproliferative disorder, plasmablastic lymphoma, primary effusion lymphoma, and extranodal NK/T cell lymphoma express PD-L1 [89, 97, 98].

The third mechanism was discovered by Kataoka and colleagues [99]. *PD-L1* 3′-untranslated region (UTR) disruption was found in a subset of DLBCL and adult T cell leukemia/lymphoma patients. The 3′-UTR disruption produces truncated PD-L1 protein, which was only found using antibody directed against the extracellular domain but not when using an antibody directed against the cytoplasmic domain. The frequency of 3′ -UTR disruption in other lymphoid neoplasms remains to be elucidated.

Lastly, PD-L1 expression can be induced by constitutive activation of the JAK/STAT pathway. In anaplastic lymphoma kinase-positive anaplastic large cell lymphoma with *NPM-ALK* rearrangement, the fusion transcript can induce PD-L1 expression mediated by activated STAT3 [100]. JAK/STAT pathway is also enhanced in DLBCL activated B cell-like (ABC) phenotype, which more commonly expresses PD-L1 compared to germinal center B cell-like (GCB) DLBCL [101]. PD-L1 expression is not generally present in other lymphoid neoplasms [102, 103].

PD-L2 expression is present in lymphoid neoplasms with abnormalities in 9p24.1/*PD-L1*/*PD-L2* [91, 104, 105]. The only exception is DLBCL, in which *PD-L2*’s expression of RNA and protein is not associated with cytogenetic abnormalities in 9p24.1 [93]. PD-L2 expression is not associated with EBV infection or 3′-UTR disruption in the *PD-L1* gene [99, 102].

Given the biology of PD-1 pathway, PD-1 expression can be best examined in the microenvironment of lymphoid neoplasms. PD-1 expression in TILs has been reported in follicular lymphoma and nodular lymphocyte predominant Hodgkin lymphoma [106, 107]. Since both neoplasms arise from germinal center B cells, it is not surprising that their microenvironments mimic their normal counterparts. Similarly, PD-1-expressing TILs are also correlated with GCB DLBCL [94]. The presence of PD-1^+^ TILs in lymphoid neoplasms could indicate cell-of-origin because PD-1^+^ TILs in follicular lymphoma (FL) and DLBCL is associated with a favorable prognosis [94, 106]. This is in contrast with solid tumors, in which presence of PD-1^+^ TILs is associated with poor prognosis [108, 109].

### Plasma cell myeloma (PCM)

*CTLA4* gene overexpression was observed in bone marrow sample from patients with PCM, suggesting additional immune-evasive signals are mediated with CTLA-4 in T cells [110]. A recent study showed that low expression of PD-1, CTLA-4, LAG-3, and TIM-3 is present on T cell clones in bone marrow and peripheral blood samples of myeloma patients, suggesting the T cells are not exhausted [111].

PD-L1 expression in myeloma cells and myeloma-propagating pre-plasma cells detected by flow cytometry has been reported in several studies [112–115]. Similar to CHL, increases in copy number of *PD-L1* correlates with PD-L1 protein expression in myeloma cells [112]. However, one study demonstrated that there was no difference regarding PD-L1 expression between normal plasma cells from healthy donors and malignant plasma cells from patients with newly diagnosed monoclonal gammopathy of undetermined significance (MGUS) or PCM [116]. Having said that, available data supports that PD-1 pathway is implicated in development of plasma cell myeloma. Bone marrow myeloma burden and serum lactate dehydrogenase level was higher in patients with PD-L1 expression in myeloma cells compared to patients without PD-L1 expression [113]. PD-L1-expressing myeloma cells are resistant to melphalan [117]. High serum soluble PD-L1 was associated with worse progression-free survival (PFS) [118]. PD-L1 expression is higher in patients with relapsed refractory plasma cell myeloma [113]. An in vitro study demonstrated that myeloma cells with expression of PD-L1 could produce exhausted T cells (CD8^+/^PD1^+/^TIM-3^+),^ instead of functional cytotoxic T cells [119]. Furthermore, co-culture of primary myeloma cells with CD4^+^/CD25−/FOXP3− T cells induced increased amount of inducible Tregs (CD4^+^/CD25^+/^FOXP3^+^) [120]. The tumor microenvironment (TME) of plasma cell myeloma is conducive to immune evasion. PD-1 overexpression was observed in T cells in patients with newly diagnosed PCM and relapsed refractory PCM [112, 116, 121]. PD-L1 expression can be induced in myeloma cells when cultivated with autologous stromal cells or human stromal cell line (HS-5) [113]. Interestingly, PD-1 expression in T cells was normalized after stem cell transplant. Additionally, PD-L1 expression is present in plasmacytoid dendritic cells or myeloid-derived suppressor cells in the TME of patients with PCM [122, 123]. PD-1 blockade showed improved survival in a myeloma murine model [119]. Unlike PD-L1, PD-L2 expression is not present in myeloma cells [112].

### Myeloid neoplasms

CLTA-4 plays a role in immune escape of AML. Using a murine myelogenous leukemia cell line (C1498) with expression of either CD80 or CD86, LaBelle et al. found that progressive tumor growth of C1498/CD80, but complete regression of C1498/CD86 after in vivo injection in naïve mice. They demonstrated that immune escape of C1498/CD80 is dependent on CTLA-4 [124]. A mouse model of relapsed AML study demonstrated that CTLA-4 blockade enhanced CTL-mediated killing of residual leukemic cells [125]. A CTLA-4 polymorphism CT60 AA genotype, located in the 3′-UTR of *CTLA4* gene, was shown to be associated with relapse in AML patients [126].

Preclinical studies demonstrated that PD-1 pathway was dysregulated in acute myeloid leukemia (AML). Murine leukemic cell C1498 shows low level PD-L1 expression when grown in vitro, but demonstrates upregulation of PD-L1 expression when grown in vivo, suggesting the microenvironment is conducive to expression of PD-L1 in leukemic cells [127]. Tregs and CD8^+^ T cells with PD-1 expression significantly increased in the liver where C1498 leukemic cells disseminate following C1498 inoculation [128]. Similar finding is also observed in the bone marrow of AML patients [129]. Tregs have suppressive effect on CD8^+^ T cell proliferation and secretion of IFN-γ from CD8^+^ T cells. However, in PD-1 knock-out (KO) mice or in wild-type mice injected with anti-PD-L1 antibody, the suppressive effect of Tregs was abrogated [128]. When C1498 leukemia cells were inoculated to PD-1 KO mice, enhanced anti-tumor response was observed with longer survival compared with C1498 inoculation to wild-type mice [127, 128]. Similar anti-tumor activity was seen with in vivo administration of anti-PD-L1 antibody to C1498-challenged wild-type mice [127, 128]. In human, mRNA expression of PD-L1 and PD-L2 is observed in many AML cell lines. However, PD-1 and CTLA-4 mRNAs were only detected in KG-1 cells [130].

Clinical data also supports dysregulated PD-1 pathway in AML. Compared to healthy individuals, PD-1 expression on T cells was significantly higher in patients with AML [131]. By quantitative polymerase chain reaction (Q-PCR), upregulation (≥twofold) of PD-L1 and PD-L2 mRNA in CD34-positive cells was observed in 36 and 12% of patients with myelodysplastic syndrome (MDS) [130]. Similarly, upregulation of abovementioned mRNAs in CD34-positive cells were seen in 25 and 33% of patients with AML, respectively. By immunohistochemistry, PD-L1 protein expression in leukemic blasts was seen in 20% of patients with MDS, chronic myelomonocytic leukemia, or AML. Of interest, upregulation of PD-L1, PD-L2, PD-1, and CLTA-4 was observed in 66% of patients with myeloid neoplasms who underwent epigenetic therapy.

In addition to PD-1 pathway and CTLA-4, another immune inhibitory molecule, TIM-3, is explored. Both human and mouse AML cells express galectin-9, a ligand of TIM-3. In a mouse model, exhausted T cells co-expressing PD-1 and TIM-3 were found, and they have reduced production of INF-γ, TNF-α, and IL-2 in reaction to their ligands-expressing AML cells. Blocking PD-1 or TIM-3 alone was not sufficient to reduce tumor burden, but combined blockade showed increased tumor rejection and improved survival [132]. The role of immune escape function of TIM-3 is also seen in AML patients as well. TIM-3 in bone marrow T cells is more frequently present in relapsed AML patients compared to those in remission or healthy donors [133].

### Role of checkpoint inhibition in hematological malignancies

#### Lymphomas

CHL is the most heavily studied lymphoid neoplasm regarding PD-1 blockade. Nivolumab (Opdivo®, Bristol-Myers Squibb) is a fully humanized IgG4 anti-PD-1 monoclonal antibody. A phase 1b study demonstrated that nivolumab has acceptable safety profile and substantial clinical activity in patients with relapsed/refractory CHL (NCT01592370) (Table 1) [134]. A subsequent phase 2 study with nivolumab (CheckMate 205 cohort B and NCT02181738) confirmed its clinical activity in relapsed/refractory CHL patients. With a median follow-up duration of 15.4 months (range 1.9–18.5 months), the objective response rate (ORR) was 68%, including complete remission (CR) and partial remission (PR) rates of 8 and 60%, respectively. 12-month overall survival and PFS rates were 94.9 and 54.6%, respectively [135–137].

**Table 1:** Notable ongoing clinical trials in hematological malignancies

Malignancies	Clinical trial #	Phase	Drug	Study description	Other name
Lymphoid neoplasm	NCT02181738	2	Nivolumab	Clinical activity of anti-PD-1 antibody in R/R CHL patients	CheckMate 205
	NCT01953692	2	Pembrolizumab	Clinical activity of anti-PD-1 antibody in R/R CHL patients	KEYNOTE-013
	NCT02857426	2	Nivolumab	Anti-PD-1 antibody in R/R PCNSL and PTL	
	NCT02576990	2	Pembrolizumab	Anti-PD-1 antibody in R/R PMBL	KEYNOTE-170
	NCT02220842	1	Atezolizumab	Anti-PD-L1 antibody in combination with anti-CD20 antibody to R/R DLBCL or FL	
Plasma cell neoplasm	NCT02036502	1	Pembrolizumab	Clinical activity of anti-PD-1, lenalidomide and low-dose dexamethasone in R/R PCM patients shown	KEYNOTE-023
	NCT02903381	2	Nivolumab	Lenalidomide, low-dose dexamethasone and anti-PD-1 antibody in smoldering PCM patients	
	NCT01592370	1	Nivolumab	Clinical activity of anti-PD-1 antibody in R/R PCM patients	
	NCT02726581	3	Nivolumab	Pomalidomide and dexamethasone with or without anti-PD-1 antibody in R/R PCM patients	CheckMate 602
	NCT02579863	3	Pembrolizumab	Pomalidomide and dexamethasone with or without anti-PD-1 antibody in treatment-naïve PCM patients	KEYNOTE-185
Myeloid neoplasms	NCT02530463	2	Nivolumab	HMA, ipilimumab, and anti-PD-1 antibody in MDS patients	
	NCT01953692	1	Pembrolizumab	Anti-PD-1 antibody in HMA-failed MDS patients	
	NCT02845297	2	Pembrolizumab	Anti-PD-1 with HMA in R/R AML patients	
	NCT02275533	2	Nivolumab	Anti-PD-1 antibody as post-remission therapy in AML patients	
	NCT02117219	1	Durvalumab	Anti-PD-L1 antibody, HMA, and tremelimumab in MDS patients	

*R/R* relapsed refractory, *PCNSL* primary central nervous system lymphoma, *PTL* primary testicular lymphoma, *PMBL* primary mediastinal large B cell lymphoma, *DLBCL* diffuse large B cell lymphoma, *FL* follicular lymphoma, *PCM* plasma cell myeloma, *HMA* hypomethylating agent, *MDS* myelodysplastic syndrome, *AML* acute myeloid leukemia

Pembrolizumab (Keytruda®, Merck & Co.) is another fully humanized IgG4 anti-PD-1 monoclonal antibody. Similar to nivolumab, pembrolizumab was shown to have manageable safety profile and favorable clinical activity in patients with relapsed/refractory CHL (NCT01953692, KEYNOTE-013) [138, 139]. The clinical activity of pembrolizumab in patients with relapsed/refractory CHL was substantiated with a multicohort phase 2 study, which included three different cohorts (KEYNOTE-087, NCT02453594). The objective response rate (ORR) was observed in 65–72% with complete remission (CR) rate of 22% in all cohorts [140, 141].

Considering underlying genetic aberrations, PMBL, PCNSL, and PTL are good candidates for PD-1 blockade. A phase 1b study (NCT01953692, KEYNOTE-013) with pembrolizumab included an independent cohort of 19 patients with relapsed/refractory primary mediastinal large B cell lymphoma. With a median follow-up of 11.3 months (range 3.4–27.4 months), the ORR was 41%, with 2 and 5 patients achieving CR and PR, respectively. On the basis of these results, a global multi-center phase 2 trial (KEYNOTE-170, NCT02576990) is ongoing [142]. An evidence-driven pilot study of nivolumab single therapy given to five patients with relapsed/refractory PCNSL and PTL found that all patients had objective radiographic responses, with four CR and one PR [143]. Encouraged by this result, a multi-institutional phase 2 single-arm trial of nivolumab in patients with relapsed/refractory PCNSL and PTL is in recruitment (NCT02857426). PD-1 blockade is also tried in patients with DLBCL, follicular lymphoma, T cell lymphoma, or mycosis fungoides/Sézary syndrome (MF/SS) with variable ORR (30–40%) (NCT01592370 and NCT02243579) [144, 145]. Other than nivolumab and pembrolizumab, other anti-PD-1 antibodies (AMP-224, BGB-A317, MEDI0680, PDR001, PF-06801591, and REGN2810) are in the lineup of immunotherapy.

Atezolizumab (Tecentriq®, Genentech) is a fully humanized IgG1 anti-PD-L1 monoclonal antibody, recently approved by the US Food and Drug Administration (FDA) for treatment of metastatic non-small cell lung cancer. Preliminary result of atezolizumab in combination with obinutumumab (anti-CD20 antibody) in patients with relapsed/refractory DLBCL or FL reported good tolerability and clinical efficacy (NCT02220842) [146]. Similar studies with atezolizumab with other agents in patients with relapsed/refractory DLBCL or FL are ongoing (NCT02729896, NCT02631577, and NCT02596971). Durvalumab (AstraZeneca) is another anti-PD-L1 antibody, approved by the FDA for treatment of bladder cancer. A few clinical trials are under way with durvalumab in patients with lymphoid neoplasms (NCT02401048, NCT02706405, and NCT02643303). Avelumab (Pfizer), CA-170 (Curis, Inc.), and BMS-936559 (Bristol-Myers Squibb) also target PD-L1, with ongoing clinical trials (NCT02603419 and NCT02812875).

Ipilimumab (Yervoy®, Bristol-Myers Squibb) and tremelimumab (Pfizer) are fully human monoclonal anti-CTLA-4 antibodies. An early pilot study of ipilimumab single therapy in patients with relapsed/refractory B cell lymphoma demonstrated low ORR (11%) [147]. Inspired by higher ORR in melanoma patients with ipilimumab and nivolumab combination therapy, ipilimumab is explored with other therapeutic agents in patients with lymphoid neoplasms (NCT01729806, NCT01896999, and NCT02254772). Ipilimumab could be an option to lymphoma patients who relapsed after allogeneic stem cell transplant. A phase 1/1b trial with ipilimumab in patients with relapsed hematologic malignancies after allogeneic stem cell transplant included 11 patients with lymphomas. Among patients treated with 10 kg/mg of ipilimumab (*n* = 22), one patient with CHL achieved a partial response and four patients (three CHLs and one cutaneous T cell lymphoma) showed a reduction in their tumor burden (NCT01822509) [148]. A premature data of ipilimumab in combination with nivolumab in 58 patients with lymphomas (NCT01592370, CheckMate 039) demonstrated that ORRs were 74, 20, and 9% of patients with CHL (*n* = 31), B cell lymphoma (*n* = 15), and T cell lymphoma (*n* = 11), respectively [149]. Similarly, tremelimumab is studied with other agents in patients with DLBCL (NCT02205333 and NCT02549651). Interestingly, ipilimumab was given to a Sézary syndrome patient with *CTLA4-CD28* rearrangement who showed a rapid clinical response [83].

### Plasma cell myeloma

A phase 1 study with nivolumab single therapy included 27 patients with relapsed/refractory PCM (NCT01592370). With the median follow-up of 65.6 weeks, stable disease was the best response in 17 (63%) patients, which lasted a median of 11.4 weeks (range 3.1–46.1 weeks) [145]. In a different arm of the same study (NCT01592370, CheckMate 039), nivolumab and ipilimumab combination therapy was tried in seven patients with relapsed/refractory PCM [149]. Only one patient (14%) showed stable disease and four patients died due to disease progression. A phase 3 study with pomalidomide and dexamethasone with or without nivolumab for patients with relapsed/refractory plasma cell myeloma is ongoing (NCT02726581, CheckMate 602). A notable phase 2 study with nivolumab, lenalidomide, and low-dose dexamethasone is underway in patients with high-risk smoldering plasma cell myeloma (NCT02903381).

A phase 1 study of pembrolizumab given in combination with lenalidomide and low-dose dexamethasone to patients with relapsed/refractory plasma cell myeloma showed responses in 20 of 40 patients (50%), including 38% of patients who were refractory to lenalidomide (KEYNOTE-023, NCT02036502) [150, 151]. Similarly, in a phase 2 study with pembrolizumab, pomalidomide, and dexamethasone given to 48 patients with relapsed/refractory plasma cell myeloma, the ORR was 56% (27 patients) including 4, 3, 6, and 14 patients with stringent CR, near CR, very good PR, and PR, respectively (NCT02289222) [152]. A similar, smaller scale study with the same regimen given to patients with relapsed/refractory plasma cell myeloma also showed clinical activity with acceptable toxicity [153]. A phase 3 study with pomalidomide and low-dose dexamethasone with or without pembrolizumab for patients with relapsed/refractory plasma cell myeloma is currently recruiting patients (NCT02576977 KEYNOTE-183) [154]. Another phase 3 study designed for patients with newly diagnosed, treatment naïve plasma cell myeloma, who are ineligible for autologous stem cell transplantation (NCT02579863, KEYNOTE-185) [155]. Similar to nivolumab, pembrolizumab is also tried to patients with intermediate- or high-risk smoldering plasma cell myeloma, but as a single therapy (NCT02603887).

There are several clinical trials with anti-PD-L1 antibodies as a single therapy or combined with others in patients with plasma cell myeloma (NCT01375842, NCT02431208, NCT02616640, NCT02685826, NCT02716805, NCT02784483, and NCT02807454), but results have not been reported yet.

Available data is limited regarding CTLA-4 blockade in patients with plasma cell myeloma. Twenty-nine patients including 6 with myeloma were enrolled in a study of ipilimumab to treat relapse after allogeneic stem cell transplant. No objective response was seen in patients with myeloma [156]. The previously described phase 1/1b trial with ipilimumab in patients with relapsed hematologic malignancies after allogeneic stem cell transplant included one patient with lung plasmacytoma, who showed a partial response without progression for more than 21 months (NCT01822509) [148]. A phase 1/2 study of combined checkpoint inhibition with nivolumab and ipilimumab in patients with plasma cell myeloma or lymphoma who are status post autologous stem cell transplant at high risk for post-transplant recurrence is underway (NCT02681302, CPIT001). A phase 1 study of tremelimumab with durvalumab is ongoing in patients with autologous stem cell transplant for plasma cell myeloma (NCT02716805).

### Myeloid neoplasms

Ipilimumab appears to be efficacious in relapsed AML patients after allogeneic stem cell transplant. The phase I/Ib study with ipilimumab (10 mg/kg) in patients with relapsed hematologic malignancies after allogeneic stem cell transplant (NCT01822509) included 16, 2, and 1 patients with AML, MDS, and myeloproliferative neoplasm, respectively. Among 22 patients treated with 10 mg of ipilimumab per kilogram, 5 patients (23%) who showed a complete including 3 with leukemia cutis, 1 with myeloid sarcoma, and 1 with AML showed a complete response. Additional four patients with AML did not achieve an objective response, but showed a reduction in the tumor burden [148].

In a phase I study, patients with high risk MDS (*n* = 11) who failed with hypomethylating agents were treated with ipilimumab monotherapy. Although objective response was not reported in any patients, disease stabilization was seen in five patients (45%) [157]. Many other clinical trials with anti-CTLA-4 antibodies are explored in patients with MDS or AML as single therapy or in combination with others (NCT01757639, NCT02117219, NCT02846376, and NCT02890329).

A single-center, phase 1b/2 study of nivolumab combined with azacitidine in patients (*n* = 51) with relapsed AML demonstrated superior survival compared to historical survival data derived from patients with relapsed AML treated with azacitidine-based salvage protocols. Among 35 patients who were evaluable for response, 6 patients (18%) with complete remission (CR) or complete remission with insufficient recovery of counts (Cri), 5 (15%) with hematologic improvement (HI), 9 (26%) had 50% bone marrow blast reduction, and 3 (9%) had stable disease. Of note, 12 patients (34%) had disease progression [158]. A preliminary result of a phase 2 study with various combinations of nivolumab, ipilimumab, and azacitidine in MDS patients (NCT02530463) was reported [159]. In the cohort of treatment-naïve MDS patients who were treated with azacitidine plus nivolumab, the ORR was 69% (9/13) with 2 CR, 5 morphologic CR and hematologic improvement (HI), and 2 HI. In the cohort of MDS patients with hypomethylating agent failure, ipilimumab single therapy showed some response (ORR 22%). However, in the same cohort, nivolumab single therapy demonstrated no response and enrollment was stopped. Preliminary result of a similar study with pembrolizumab (KEYNOTE-013, NCT01953692) in patients with MDS who failed with hypomethylating agents was also reported. The ORR was 4% (1/27) with no CR and 1 PR [160]. Other clinical trials of anti-PD-1 antibody in combination with hypomethylating agent(s) in patients with MDS or AML patients are ongoing (NCT02845297 and NCT02599649).

Anti-PD-1 antibody can be explored with chemotherapeutic agents in patients with AML. NCT02464657 and NCT02768792 are such studies. In another angle, PD-1 blockade can be tried in AML patients who are in remission (NCT02275533, NCT02532231, and NCT02708641). A phase 2 study of pembrolizumab in patients with non-favorable risk AML who underwent lymphodepletion with fludarabine and melphalan followed by autologous transplantation will be interesting (NCT02771197).

Among anti-PD-L1 antibodies, durvalumab is actively studied in patients with MDS or AML. A phase 2 study with oral azacitidine with durvalumab in patients with MDS who failed with hypomethylating agents is underway (NCT02281084). A similar phase 2 study, but with subcutaneous azacitidine in combination with durvalumab in treatment-naïve MDS or elderly (≥65 years) AML patients is also underway (NCT02775903). A phase 1 study with durvalumab single therapy or in combination with tremelimumab with or without azacitidine to patients with MDS is ongoing (NCT02117219).

### Side effects of checkpoint therapy

Checkpoint inhibitors, like any other drugs, do not provide benefits to patients without risks. Immune-related adverse events (irAEs) are a spectrum of side effects including gastrointestinal, dermatologic, hepatic, or endocrine events. It is usually recommended for patients with grade 2 irAEs to withhold checkpoint inhibitor transiently. For patients with grade 3 or higher irAEs, checkpoint inhibitor should be stopped and treated with systemic corticosteroids (1 to 2 mg/kg or equivalent) daily. Other immune modulatory agents such as Infliximab can be considered for patients without improvement with steroids [161].

In general, IrAEs with anti-PD-1 antibodies are less common than anti-CTLA-4 antibody. In 298 melanoma patients treated with ipilimumab (3 mg/kg), irAEs of any grade were reported in 85% of patients [162]. Grade 3 or higher irAEs are seen in 112 patients (38%), diarrhea being the most common irAE followed by hepatotoxicity, dermatitis, hypophysitis, and uveitis. Approximately 1/3 of patients were treated with systemic corticosteroids, but it did not affect OS or time-to-treatment failure indicating generous use of corticosteroid for irAEs. In a pooled analysis of 576 melanoma patients treated with nivolumab (3 mg/kg), 71% of patients suffered irAEs of any grade [163]. Grade 3 or higher irAEs were seen in 57 (10%) of patients including neurologic AEs, autoimmune neuropathy, central demyelination, Guillain-Barré syndrome, and involuntary muscle contractions. Similar to ipilimumab, management of irAEs with systemic corticosteroids did not affect treatment response of nivolumab. Grade 3 or higher irAEs were more common in melanoma patients who were treated with combined nivolumab and ipilimumab compared with those treated with either ipilimumab or nivolumab single therapy (55, 25, and 16%, respectively) [164, 165].

### Biomarkers related to checkpoint inhibitor therapy

Although the clinical efficacy of anti-PD-1 therapy has been proven, not all cancer types respond to anti-PD-1 therapy. In solid tumors, immunologically responsive tumors vs. immunologically ignorant tumors are recognized based on immune cell infiltration in the TME. The former tend to be seen with numerous T cells in the TME (inflamed tumors) and to have a high mutational load in tandem with neoantigens with higher quantity. Immunologically responsive tumors are more likely responsive to anti-PD-1 therapy [166]. However, anti-PD-l therapy is not effective in all patients with responsive tumors and even in those with response, delayed, or mixed tumor regression can be seen [167]. Furthermore, manipulation of immune checkpoints with anti-PD-1 agents not uncommonly causes irAEs. Therefore, biomarkers to selectively identify best candidates are much needed.

Several methods are currently available (Table 2). PD-L1 expression in tumor cells assessed by immunohistochemistry has been associated with better response to anti-PD-1 therapy in solid tumors as well as in CHL [167, 168]. However, there are different types of clones for PD-L1 immunohistochemical antibodies and standardization has not been achieved. Similarly, the serum level of soluble PD-L1 measured by enzyme-linked immunosorbent assay (ELISA) can be a potential predictive biomarker in patients with DLBCL or PCM [118, 169]. However, these patients were treated with conventional chemotherapy, so investigation targeting the PD-1 pathway must be conducted. TILs, particularly with PD-L1 expression, were associated with higher response to PD-1-targeting therapy in patients with solid tumors [5, 170]. However, data are not available regarding TILs with PD-L1 expression in patients with lymphoma. Assessment of dynamics in immune cell profiles in the TME of biopsy samples using immunohistochemistry at different time points during the treatment schedule sheds light on the prediction of response. Chen et al. has demonstrated that immune cell profiles early in treatment, not before treatment, are predictive of treatment response. They also showed that gene expression profiling using a 795-gene NanoString panel recapitulates the result [171]. Immune cell profiles can also be evaluated with peripheral blood using flow cytometry [172, 173].

**Table 2:** Potential predictive and prognostic biomarker evaluation and technologies

Technology	Target cells/tissue	Purpose	Reference
Immunohistochemistry	FFPE tissue	Analysis of protein expression in tumor cells Immune cell profiling in TME	[11, 157]
Flow cytometry	Blood, fresh/frozen tumor tissue	Analysis of different subsets of immune cells	[141]
ELISA	Blood	Analysis of cytokine Chemokine and antibodies against tumor-specific antigen	[162]
Enzyme-linked immunospot	Blood	Quantification of T or B cells, Analysis of cytokine and chemokine	[158]
Protein microarray	Blood	Analysis of antibody signature	[159]
Gene expression profiling	Blood, fresh/frozen/FFPE tumor tissue	Analysis of gene signatures in tumor/immune cells	[145]
TCR deep sequencing	Blood	T cell receptor profiling	[160]
NGS (WES, RNA-seq)	Fresh/FFPE tissue	Mutational load Prediction of immunogenecity of tumor neoantigens	[92, 143]
Epigenomics	Blood, fresh/frozen/FFPE tumor tissue	Analysis of immune cell specific epigenetic changes	[161]

*FFPE* formalin-fixed, paraffin-embedded, *TME* tumor microenvironment, *ELISA* enzyme-linked immunosorbent assay, *NGS* next-generation sequencing, *WES* whole exome sequencing

The higher the mutational load in cancer cells, the more neoantigens are produced in them. Neoantigens generally have high antigenicity, which attracts immune cells (inflamed tumors). High mutational load is associated with a better response to anti-PD-1 therapy [174]. However, the number of mutations in cancer cells does not directly correlate with the production of high-quality neoantigens. A computational genomic tool has been developed to predict immunogenicity of mutagen-derived neoantigens or cancer germline antigens and their binding affinity to immune cells. It can further provide prediction of response when treated with anti-PD-1 or anti-CTLA-4 agents [175, 176]. The computational genomic tool was shown to be feasible with solid tumor, yet applicability of mutational load as a biomarker in lymphomas is questionable due to lack of data [174, 177].

Not all of abovementioned methods can be applicable to hematologic malignancies because most data was driven from patients with solid tumors or solid tumor models. However, efforts to detect intrinsic overexpression of PD-L1 or PD-L2 are recommended to identify possible good responders to anti-PD-1/PD-L1 therapy. That is, immunohistochemistry for PD-L1/PD-L2 protein expression, chromosome analysis, or fluorescence in situ hybridization to detect aberrations in 9p24.1/PD-L1/PD-L2 locus or RT-PCR to detect gene rearrangements involving *PD-L1* or *PD-L2* could be a minimum step.

## Conclusions

Inhibitory molecules such as PD-1, CTLA-4, LAG-3, or TIM-3 play a role to keep a balance in immune function. However, many cancers exploit such molecules to escape immune surveillance. Accumulating data support that their functions are dysregulated in lymphoid neoplasms, plasma cell myeloma, myelodysplastic syndrome, and acute myeloid leukemia. Clinical trials demonstrated that PD-1 blockade is an attractive way to reinstate host’s immune function in lymphoid neoplasms, particularly classical Hodgkin lymphoma. PD-1 blockade as a single therapy or in combination with other immune checkpoint inhibitors are explored in other hematologic cancers. Of note, not all patients respond to immune checkpoint inhibitors. Therefore, the need to identify best candidates who would have excellent response to checkpoint inhibitors is high. Several possible biomarkers are available, but consensus has not been made and pursuit to discover the best biomarker is ongoing.

## Abbreviations

ABC: Activated B cell-like
ALK: Anaplastic lymphoma kinase
AML: Acute myeloid leukemia
AP-1: Activator protein 1
APC: Antigen-presenting cell
BAT3: HLA-B associated transcript 3
BTLA: B- and T-lymphocyte attenuator
CDK4: Cyclin-dependent kinase 4
CDK6: Cyclin-dependent kinase 6
CHL: Classical Hodgkin lymphoma
CR: Complete remission
CTLA-4: Cytotoxic T-lymphocyte antigen-4
DLBCL, NOS: Diffuse large B-cell lymphoma, not otherwise specified
EBV: Epstein-Barr virus
ELISA: Enzyme-linked immunosorbent assay
ERK: Extracellular signal-regulated kinase-1
FDA: US Food and Drug Administration
FL: Follicular lymphoma
FOXP3: Forkhead box P3
GCB: Germinal center B cell-like
GM-CSF: Granulocyte macrophage colony-stimulating factor
HAVCR2: Hepatitis A virus cellular receptor 2
HI: Hematologic improvement
HMGB1: High mobility group protein B1
IFN-γ: Interferon gamma
IL-2: Interleukin-2
ITIM: Immune receptor tyrosine-based inhibitory motif
ITSM: Immunoreceptor tyrosine-based switch motif
JAK/STAT: Janus kinase/signal transducer and activator of transcription
JNK: c-Jun NH2-terminal kinase
KO: Knock-out
LAG-3: Lymphocyte activation gene 3
LMP1: EBV latent membrane protein 1
LSECtin: Liver and lymph node sinusoidal endothelial cell C-type lectin
MAPK: Microtubule-associated protein kinase
MDS: Myelodysplastic syndrome
MF/SS: Mycosis fungoides/Sézary syndrome
MGUS: Monoclonal gammopathy of undetermined significance
MHC: Major histocompatibility complex
NFAT: Nuclear factor of activated T cells
ORR: Objective response rate
PCM: Plasma cell myeloma
PCNSL: Primary central nervous system lymphoma
PD-1: Programmed death-1
PD-L1: Programmed death ligand-1
PD-L2: Programmed death ligand-2
PFS: Progression-free survival
PI3K: Phosphatidylinositol 3-kinase
PKC: Protein kinase C
PMBL: Primary mediastinal large B-cell lymphoma
PP2A: Protein phosphatase 2
PR: Partial remission
PTL: Primary testicular lymphoma
Q-PCR: Quantitative polymerase chain reaction
SHP: Src homology 2 domain-containing phosphatase
TCHRBCL: T cell rich, histiocyte-rich large B cell lymphoma
TCR: T cell receptor
TGF-β: Transforming growth factor beta
TIGIT: T cell immunoreceptor with immunoglobulin and ITIM domains
TIL: Tumor-infiltrating lymphocyte
TIM-3: T cell immunoglobulin and mucin domain-containing protein-3
TME: Tumor microenvironment
Treg: Regulatory T cells
UTR: Untranslated region
ZAP70: Zeta-chain-associated protein kinase 70
